# Multimodal Late-Fusion of Radiomics, Clinical Data, and Demographics Enhances Personalized Survival Prediction in NSCLC

**DOI:** 10.3390/cancers18091407

**Published:** 2026-04-29

**Authors:** Zarindokht Helforoush, Mohamed Jaber, Nezamoddin N. Kachouie

**Affiliations:** 1 Department of Mathematics and Systems Engineering, Florida Institute of Technology, Melbourne, FL 32901, USA; zhelforoush2022@my.fit.edu (Z.H.); mjaber@fit.edu (M.J.); 2 Department of Electrical Engineering and Computer Science, Florida Institute of Technology, Melbourne, FL 32901, USA

**Keywords:** radiomics, non-small cell lung cancer, multimodal fusion, group lasso, random survival forest, prognostic modeling, risk stratification

## Abstract

**Backgrounds/Objectives:** Non-small cell lung cancer (NSCLC) exhibits substantial prognostic heterogeneity that is not fully captured by conventional anatomical staging, highlighting the need for individualized risk assessment. Radiomics enables non-invasive characterization of tumor phenotype, yet high dimensionality and inter-feature correlations often limit model stability and interpretability. **Methods**: To address these challenges, we developed a multimodal late-fusion framework integrating radiomic, clinical, and demographic information to predict patient-specific absolute risk in the Lung1 cohort (N = 398). Radomic features (N = 107) were extracted from primary tumor volumes and refined using a Group Lasso–penalized Cox model, preserving biological coherence and producing a parsimonious imaging signature. This signature was combined with clinical and demographic variables using five different late-fusion strategies: weighted averaging, Cox regression, logistic stacking, Random Survival Forests (RSF), and XGBoost. Model performance was evaluated using 5-fold cross-validation based on discrimination, calibration, and risk stratification metrics. **Results**: Using 5-fold cross validation, the radiomics-only model outperformed conventional clinical staging in patients’ risk prediction (C-index 0.5717 vs. 0.5350) and accuracy, demonstrating the prognostic value of imaging biomarkers. All fusion strategies improved risk prediction performance, with the Cox fusion model slightly better than other fusion methods with C-index of 0.58, time-dependent AUC of 0.60, and the distinct risk stratification with log-rank χ^2^ of 22.85. **Conclusions**: These findings suggest that multimodal late fusion may provide robust and interpretable risk estimates with potential clinical relevance, supporting personalized risk prediction for informed decision-making in NSCLC.

## Simple Summary

Predicting survival in non-small cell lung cancer (NSCLC) is challenging because patients with the same disease stage can have very different outcomes. This study developed a method that combines quantitative imaging features from CT scans (radiomics) with clinical and demographic information to better estimate individual patient risk. Using a dataset of 398 patients, our results show that combining data from multiple sources improves prediction accuracy compared with using imaging or clinical information alone. In particular, a Cox fusion model provided the most precise risk estimates and clearly separated patients into low-, medium-, and high-risk groups, even identifying high-risk individuals in early-stage disease and low-risk individuals in advanced-stage disease. This approach provides a practical framework for personalized risk assessment and could help guide clinical decision-making in NSCLC.

## 1. Introduction

Lung cancer remains the leading cause of cancer-related mortality worldwide, accounting for approximately one in five cancer deaths annually, with non-small cell lung cancer (NSCLC) representing nearly 85% of all cases [1,2]. Despite substantial advances in diagnostic imaging, surgical techniques, and systemic therapies, considerable variability in clinical outcomes persists, even among patients with the same disease stage. This pronounced heterogeneity highlights the limitations of population-based prognostic frameworks and emphasizes the need for more individualized risk assessment to enable precise patient stratification and support informed clinical decision-making. In routine clinical practice, prognostic stratification in lung cancer is primarily guided by anatomical staging systems, most notably the TNM classification. Although these systems provide essential population-level guidance, they are inherently limited in their ability to capture inter-patient biological variability and intratumoral heterogeneity, resulting in suboptimal risk discrimination within stage-defined groups [3,4]. Consequently, there is increasing interest in prognostic models that incorporate complementary patient-specific information beyond conventional staging variables.

Radiomics has emerged as a powerful paradigm for extracting high-throughput quantitative descriptors from routinely acquired medical images, enabling noninvasive characterization of tumor phenotype, heterogeneity, and spatial organization [5,6,7]. In lung cancer, radiomics-derived features have demonstrated prognostic value beyond traditional clinical variables. However, the high dimensionality, strong multicollinearity, and grouped structure of radiomic features present substantial methodological challenges, particularly in survival analysis with moderate sample sizes. Without appropriate regularization, radiomics-based models are prone to instability, overfitting, limited generalizability, and reduced interpretability [8,9]. To address these challenges, structured feature selection methods that explicitly account for the grouped nature of radiomics features have gained increasing attention. Group-based penalization approaches enable simultaneous feature selection and coefficient estimation at the level of predefined feature families, promoting model parsimony while preserving biological and methodological coherence [10,11,12].

Beyond imaging-derived biomarkers, demographic variables such as age and sex, along with clinical staging parameters including overall stage and clinical stage remain among the most robust predictors of lung cancer survival. These modalities capture complementary aspects of disease and host characteristics: radiomics reflect tumor-level phenotypic attributes, clinical staging summarizes anatomical disease burden, and demographic factors encode host-related risks. Integrating these heterogeneous data sources therefore represents a rational strategy for improving individualized prognostic modeling. Multimodal survival modeling can be implemented through early fusion, which combines raw features across modalities, or late fusion, which integrates modality-specific predictions. Early fusion approaches may be hindered by high dimensionality and heterogeneous feature scales, particularly when high-dimensional radiomic features are integrated with low-dimensional clinical variables in moderate-sized cohorts [13,14,15]. In contrast, late fusion provides a more robust and clinically intuitive alternative by preserving modality-specific risk estimates and reducing the risk of overparameterization [15,16,17]. Importantly, references [15,16,17] provide general background on multimodal fusion, whereas more directly related prognostic studies in lung cancer have used multimodal radiomics with ensemble survival modeling or explicitly compared multiple fusion strategies [13,14]. Fusion approaches have demonstrated prognostic improvements in risk analysis [18] supporting the value of multimodal integration.

Cox proportional hazards models naturally estimate relative risk, however absolute risk predictions at clinically meaningful time horizons are more directly interpretable and actionable in clinical practice. Absolute risk supports patient counseling, treatment prioritization, and risk-based stratification, and is increasingly recognized as essential for clinically useful prognostic tools [19,20].

In this study, we propose a comprehensive multimodal late-fusion framework designed to address the challenges of high-dimensional radiomic data and enhance individualized risk prediction in NSCLC. Using structured Group Lasso regularization, we first derive a parsimonious and biologically interpretable imaging signature that preserves feature-family coherence while mitigating overfitting. We then systematically evaluate the integration of this radiomic signature with clinical and demographic predictors using five late-fusion strategies: Cox regression, weighted averaging, logistic stacking, Random Survival Forests and XGBoost. Unlike prior studies that adopted a single ensemble or fusion model, the proposed framework enables direct comparison of multiple linear and non-linear late-fusion methods, while preserving modality-specific interpretability [13,14]. This framework enables rigorous assessment of linear and non-linear integration methods in capturing cross-modal interactions. Ultimately, the proposed approach seeks to translate heterogeneous multimodal data into precise, patient-specific absolute risk estimates, thereby refining prognostic stratification and supporting more personalized clinical decision-making.

## 2. Data Description

This study utilized the Lung1 cohort available through the Cancer Imaging Archive (TCIA), a well-curated and widely adopted public dataset for radiomics-based prognostic modeling in non-small cell lung cancer (NSCLC). The dataset comprises 107 quantitative radiomic features together with corresponding demographic variables, clinical staging information, and overall survival outcomes. Radiomic features were derived exclusively from primary tumor regions provided within the Lung1 dataset, ensuring methodological consistency with prior benchmark studies while minimizing variability associated with heterogeneous segmentation practices. Tumor segmentations were publicly available through TCIA; therefore, no additional manual delineation or automated re-segmentation procedures were performed. All radiomic features were extracted in three dimensions using voxel-based analysis to comprehensively characterize volumetric tumor properties. Feature extraction was conducted using the PyRadiomics software library (version 3.0) in accordance with standardized feature definitions and computational protocols established by the Image Biomarker Standardization Initiative (IBSI), thereby promoting reproducibility and facilitating cross-study comparability. To enable patient-level survival analysis, cases with multiple tumor lesions were harmonized by aggregating lesion-specific information into a unified patient-level representation. Secondary lesions were incorporated as an aggregate covariate reflecting the total number of tumors per patient, consistent with established practices in radiomics-based prognostic modeling [5]. A total of 422 patients with NSCLC were initially identified. After excluding 24 patients with incomplete records, the final study population consisted of 398 patients. The study population included individuals diagnosed with stages I, II, IIIa, and IIIb lung cancer. Stages IIIa and IIIb were distinguished based on tumor anatomical location and the extent and pattern of regional lymph node involvement, capturing clinically meaningful differences in disease burden and prognosis [21,22]. Baseline demographic and clinical characteristics of the final cohort are summarized in Table 1. Radiomic features were organized into seven predefined and conceptually distinct categories, including First-Order Statistics, Shape Descriptors, and five texture-based feature families: Gray-Level Co-occurrence Matrix (GLCM), Gray-Level Run Length Matrix (GLRLM), Gray-Level Size Zone Matrix (GLSZM), Gray-Level Dependence Matrix (GLDM), and Neighborhood Gray-Tone Difference Matrix (NGTDM) [5,23].

Radiomic features were organized according to established radiomic taxonomy to enable biologically interpretable modeling within a group-penalized regression framework. First-order statistical features describe the distribution of voxel intensity values within the tumor volume without accounting for spatial relationships, thereby capturing global intensity characteristics such as central tendency, dispersion, and distributional shape [24]. In contrast, shape descriptors quantify geometric and morphological properties of the tumor, including size, surface irregularity, sphericity, and compactness attributes that have been associated with tumor aggressiveness and growth dynamics [25]. Texture-based feature classes provide higher-order characterization of intratumoral heterogeneity by quantifying spatial relationships and structural patterns within imaging data. GLCM features measure the frequency of co-occurring voxel intensity pairs at predefined spatial offsets, capturing textural regularity and local contrast patterns [24]. GLRLM features quantify the length of consecutive runs of voxels sharing identical gray-level intensities, reflecting differences between coarse and fine structural organization. GLSZM features assess the distribution of homogeneous intensity zones irrespective of spatial orientation and are particularly sensitive to regional uniformity and heterogeneity within the tumor volume. GLDM features evaluate local voxel dependencies by measuring the number of neighboring voxels with similar intensities, providing insight into texture complexity and structural granularity [26]. Finally, NGTDM features characterize fine-scale textural variation by quantifying the difference between a voxel’s intensity and the mean intensity of its surrounding neighborhood, thereby reflecting local contrast and edge definition [24]. Collectively, these radiomic biomarkers provide a comprehensive and non-invasive characterization of tumor phenotype by integrating information related to intensity distribution, tumor geometry, and spatial heterogeneity. Building on prior evidence linking radiomic heterogeneity to clinical outcomes in lung cancer, this study develops a Cox proportional hazard–based risk stratification framework that jointly incorporates clinical variables, demographic factors, and imaging-derived features. The developed model stratifies patients into low-, medium-, and high-risk groups, providing a refined prognostic framework that extends beyond conventional staging systems and supports more individualized risk assessment and may help inform clinical decision-making.

## 3. Methods

The methodological framework of this study was designed to address the challenges of high-dimensional, multimodal survival analysis while maintaining clinical interpretability. Time-to-event outcomes were modeled using Cox proportional hazards regression and its penalized extensions, including a Group Lasso formulation to enable structured feature selection among correlated radiomic predictors. In parallel, flexible machine-learning and ensemble (late-fusion) strategies, including Cox regression, weighted averaging, logistic stacking, Random Survival Forests, and XGBoost, were employed to capture complementary prognostic information across data modalities. In these fusion approaches, three modality-specific risk scores—derived from clinical, demographic, and radiomic models—were used as input predictors in meta-models to enable integrated risk estimation. Model-derived survival probabilities at a predefined time horizon were transformed into absolute risk estimates to facilitate data-driven risk group construction. The performance of all models was evaluated both on the full dataset and through five-fold cross-validation, with the cross-validated metrics reported as the primary performance estimates. Stratification performance was subsequently evaluated using log-rank statistics. Collectively, this integrated framework supports robust prognostic modeling and enables systematic comparison of competing survival analysis strategies.

### 3.1. Cox Regression and Penalized Cox Proportional Hazards Model

Survival analysis was conducted using the Cox proportional hazards (PH) model to quantify associations between patient-specific covariates and time-to-event outcomes. The Cox model is a semi-parametric approach in which the hazard function is expressed as the product of an unspecified baseline hazard and an exponential function of covariates, thereby avoiding the need to assume a specific parametric form for the underlying hazard distribution. This flexibility, combined with the direct interpretability of regression coefficients as hazard ratios, has established the Cox model as the standard analytical tool in clinical and biomedical survival research. The Cox PH model relies on several key assumptions, including independence of survival times, proportional hazards throughout follow-up, and a linear relationship between covariates and the logarithm of the hazard function [27]. Violations of these assumptions can adversely affect both inference and predictive performance, particularly in complex, high-dimensional datasets such as those encountered in radiomics. Given the high dimensionality, strong multicollinearity, and grouped structure characteristic of radiomic feature spaces, penalized extensions of the Cox model were employed. Penalized Cox regression incorporates regularization terms into the partial likelihood function to constrain coefficient magnitudes and control model complexity. This approach enables simultaneous coefficient shrinkage and variable selection, which is essential when the number of predictors approaches or exceeds the number of observed events. By reducing variance and mitigating overfitting, penalization improves numerical stability and enhances out-of-sample generalizability while preserving the interpretability and clinical relevance of the Cox modeling framework [28]. These properties make penalized Cox models particularly well suited for radiomics-based prognostic analysis and align with methodological expectations for translational oncology. particularly appropriate for radiomics-driven prognostic modeling.

### 3.2. Evaluation Metrics

Model performance was primarily evaluated in terms of discrimination, defined as the ability of each model to accurately rank patients according to their risk of experiencing the event of interest. Discriminatory performance was quantified using the concordance index (C-index), which represents the proportion of all comparable patient pairs for which predicted risk is consistent with observed survival outcomes [29]. A C-index of 0.5 indicates no predictive discrimination beyond random chance, whereas a value of 1.0 reflects perfect concordance between predicted and observed event ordering. To account for potential temporal variation in prognostic relevance during follow-up, model discrimination was further assessed using time-dependent receiver operating characteristic (ROC) analysis. Time-dependent area under the ROC curve (AUC) values were calculated at prespecified time horizons, enabling appropriate handling of right-censored survival data while providing a dynamic evaluation of predictive accuracy over time [30]. In contrast to the C-index, which summarizes global ranking performance across the entire follow-up period, time-dependent AUC quantifies the model’s ability to distinguish between patients who experience the event by a given time point and those who remain event-free. Together, these complementary metrics provide a comprehensive assessment of prognostic discrimination from both global and time-specific perspectives. In addition to discrimination, overall predictive accuracy was evaluated using the Brier score, which measures the mean squared difference between predicted survival probabilities and observed outcomes, thereby jointly reflecting discrimination and calibration. Lower Brier scores indicate improved predictive performance and more accurate probability estimation [31].

### 3.3. Group Lasso Framework

**Figure 1:**
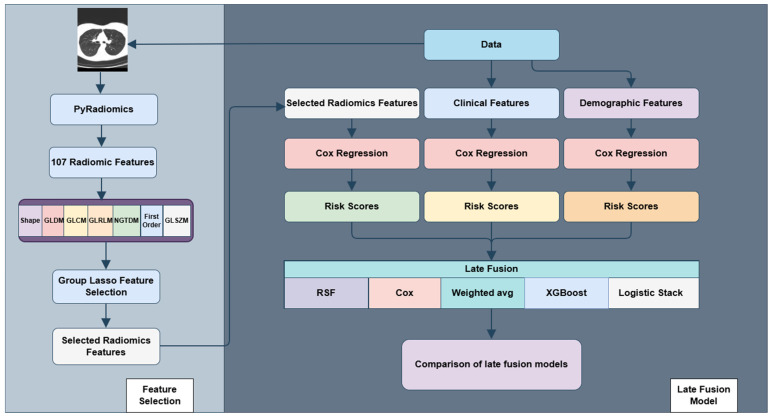
The workflow of this study starts with feature extraction from CT images using PyRadiomics followed by feature selection using Group Lasso and concluded by risk stratification by late fusion. Arrows indicate workflow progression.

Group Lasso-based regularization extends conventional penalization methods by performing variable selection and coefficient estimation at the level of predefined feature groups rather than individual predictors. This strategy is particularly well suited for high-dimensional survival analysis, where variables frequently exhibit natural clustering such as radiomic feature families derived from shared computational definitions or textural principles. Within the group survival modeling (grpsurv) framework, the generalized group Lasso (grLasso) provides a structured and principled mechanism for incorporating grouped predictors into a Cox proportional hazards model. As illustrated in Figure 1, the analytical workflow begins with radiomic feature extraction from CT images using PyRadiomics (version 3.0), followed by group-based penalized Cox modeling for feature selection, derivation of patient-specific risk scores, risk stratification, and comprehensive performance evaluation. The grLasso penalty applies an ℓ_2_ norm within each predefined group and an ℓ_1_ norm across groups. This hierarchical penalty structure promotes sparsity at the group level, encouraging entire radiomic feature families to be retained or excluded collectively, thereby preserving interpretability and biological coherence.

A key advantage of grLasso is its ability to incorporate data-adaptive group weights. This flexibility accommodates heterogeneity in group size and signal strength, reducing selection bias toward larger groups and promoting balanced treatment across feature families. By enforcing structured sparsity while maintaining controlled shrinkage within selected groups, grLasso achieves an effective trade-off between dimensionality reduction, estimation stability, and clinical interpretability. In the context of high-dimensional radiomics-based survival modeling, this framework mitigates multicollinearity, limits overfitting, and aligns model construction with the intrinsic organization of imaging-derived features. Consequently, it supports robust, reproducible, and clinically meaningful prognostic modeling consistent with current standards for translational oncology research [32].

### 3.4. Random Survival Forest

Random Survival Forests (RSF) constitute a nonparametric ensemble learning method for right-censored time-to-event data, extending the random forest framework originally introduced by Leo Breiman to the domain of survival analysis Ishwaran [33,34]. RSF builds an ensemble of survival trees using bootstrap aggregation (bagging). For each tree, a bootstrap sample of the data is drawn, and at every node a random subset of predictors is evaluated. Candidate splits are selected to maximize survival separation between daughter nodes, most commonly using log-rank test statistics. Within each terminal node, survival functions are estimated nonparametrically using estimators such as the Kaplan–Meier or Nelson–Aalen estimator. Ensemble-level predictions are then obtained by averaging survival estimates across all trees, yielding stable and robust predictions. A key advantage of RSF is that it does not assume proportional hazards, allowing it to accommodate complex nonlinear relationships, high-order interactions, and non-additive effects among predictors. RSF produces patient-specific survival functions as well as time-dependent risk estimates, enabling flexible modeling of heterogeneous prognostic patterns over time. These properties make RSF particularly well suited for multimodal prognostic modeling, where interactions among clinical, demographic, and imaging-derived variables may be intricate and not well captured by traditional linear survival models [33,34]. Hyperparameter tuning for the Random Survival Forest was performed using a random grid search of over 50 candidate configurations. The candidate grid included mtry values of 1 and 2 (number of candidate variables randomly sampled and examined for splitting at each node of a tree), node size values of 5, 8, 10, 15, 20, 25, and 30, number of tree values of 500, 750, 1000, and 1500, and number of split values of 1, 3, 5, and 10. These tuning steps were carried out within the cross-validation framework to support a fair and robust evaluation of model performance.

### 3.5. Weighted Averaging Ensemble

Weighted averaging is a linear ensemble strategy that combines modality-specific predictions into a single composite risk estimate. Predictions generated by each modality-specific model are aggregated using either equal weights or data-driven optimized weights, allowing flexible control over their relative contributions to the final prediction. This linear aggregation framework has strong theoretical foundations in ensemble learning and is known to reduce prediction variance while enhancing generalization performance. In the context of survival analysis, the aggregated prediction is typically calibrated at a prespecified time horizon to yield clinically interpretable absolute risk estimates. Because the method preserves the structure of individual model outputs, it enables transparent integration of heterogeneous data sources while maintaining interpretability, an important consideration for clinical applications. Weighted averaging is particularly effective when individual models capture complementary aspects of prognosis, as the ensemble can leverage diverse information without introducing additional model complexity or strong parametric assumptions. Consequently, it provides a stable, interpretable, and computationally efficient late-fusion strategy for multimodal survival modeling [35,36].

### 3.6. Logistic Stacking

Stacking, or stacked generalization, is a supervised ensemble methodology that combines predictions from multiple base models using a secondary model (meta-learner) trained to optimize overall predictive performance. In logistic stacking, modality-specific predictions serve as inputs to a logistic regression meta-model, which estimates data-driven weights that reflect the relative contribution of each modality to the final prediction. For survival analysis, logistic stacking is commonly implemented at a fixed prediction horizon by transforming the time-to-event outcome into a binary endpoint (e.g., event occurrence by a specified time). This formulation enables direct estimation of calibrated risk probabilities while appropriately incorporating right-censored data through prior survival modeling. By learning weights from the data rather than assigning them a priori, logistic stacking can adapt to differences in predictive strength across modalities. Compared with simple averaging, logistic stacking offers greater flexibility by accounting for correlations among base learners and capturing complementary prognostic information while retaining interpretability and computational efficiency. These characteristics make it a practical and robust late-fusion strategy for multimodal prognostic modeling in translational oncology research [37]. Regularization in the logistic stacking layer was implemented within a nested cross-validation framework using elastic net penalties, with the mixing parameter alpha tuned over the values 0, 0.25, 0.5, 0.75, and 1.

### 3.7. XGBoost Stacking

XGBoost stacking combines XGBoost’s optimized gradient boosting where decision trees are sequentially built to minimize loss via second-order approximations and regularization with stacked generalization, using multiple XGBoost (released version 3.1.0) base models to generate out-of-fold predictions via cross-validation, which feed into a meta-learner (e.g., logistic regression or another XGBoost) for final predictions, enhancing generalization by leveraging diverse model strengths [38]. For the XGBoost-based stacking model, hyperparameters were tuned within a nested cross-validation via grid search across maximum depth values of 1, 2, and 3 and learning rate values of 0.03, 0.05, and 0.1 [37,38].

### 3.8. Risk Group Construction

Patient risk stratification was performed using model-derived survival predictions obtained from the penalized Cox proportional hazards model fitted at the optimal regularization level via five-fold cross-validation. Individual survival probabilities were estimated at the 1.5-year landmark, corresponding approximately to the median follow-up time of the cohort. Absolute risk at this time point was calculated as one minus the predicted survival probability, providing the cumulative event probability used for subsequent assignment to prognostic risk groups. These predicted absolute risk scores formed the basis for defining clinically interpretable risk categories. Within each training fold of the cross-validation procedure, continuous model-derived risk scores were discretized into three ordered prognostic groups (low, medium, and high risk) using a data-driven, outcome-guided procedure designed to balance statistical separation with group stability. Candidate cutoff pairs were generated across the central portion of the risk-score distribution to avoid extreme thresholds influenced by outliers. For each candidate pair, patients were partitioned into three risk groups, and differences in time-to-event outcomes across groups were assessed using the log-rank statistics. The optimal cutoffs—defined as those maximizing between-group separation in survival while satisfying minimum group-size constraints—were identified separately within each training fold; the final cutoffs (Cutoff_1_ and Cutoff_2_) were then computed as the averages across all folds and applied to the cross-validated predicted risk scores on held-out test folds. This stratification approach explicitly incorporates survival information into the definition of risk categories, rather than relying on arbitrary quantiles or purely distribution-based thresholds. By enforcing monotonic ordering of risk groups and minimum prevalence criteria, the method ensures both discriminative separation and clinical plausibility of the resulting categories. Overall, this framework produces prognostic groups that are well separated with respect to survival and adequately populated for downstream analyses, while providing a structured translation from continuous prognostic scores to clinically relevant risk stratification.

### 3.9. Calibration Plots

Model calibration was evaluated using calibration plots to assess the reliability of predicted survival probabilities against observed outcomes. In these plots, patients are stratified into risk quantiles based on model-derived probabilities at a fixed time horizon, with mean predicted probabilities plotted against corresponding Kaplan–Meier estimates of observed event rates; a well-calibrated model aligns closely with the 45° reference line. Quantitative metrics supplemented this visual assessment, including the calibration slope (ideally near 1, indicating appropriate predictions), calibration intercept (ideally near 0, indicating minimal bias) [39].

## 4. Results

This section presents a comprehensive evaluation of the proposed survival modeling framework within the NSCLC cohort. Results are organized into three parts: (1) identification of radiomic features through group-based penalized Cox modeling, (2) comparison of predictive performance using discrimination and calibration metrics and (3) formation and evaluation of model-derived prognostic risk groups using unimodal and late-fusion approaches, emphasizing the added value of multimodal integration for risk stratification and potential clinical relevance.

### 4.1. Selected Features

**Figure 2:**
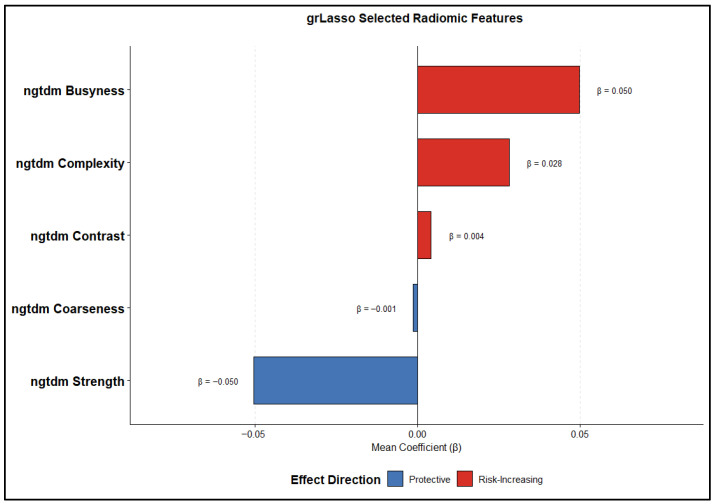
Feature importance and effect direction of radiomic predictors.

Given the high dimensionality of radiomic features and the strong correlations inherent to quantitative imaging descriptors, a group-based penalized Cox proportional hazards framework was employed for robust feature selection, mitigating redundancy and overfitting. Radiomic features were organized into biologically and mathematically coherent families corresponding to predefined radiomic categories. To enhance robustness, feature selection and coefficient estimation were performed within a nested 5-fold cross-validation framework, in which model tuning was conducted on the training folds only and predictor standardization was performed using fold-specific training-set to prevent information leakage. Feature selection and coefficient estimation were conducted using a group Lasso–penalized Cox model (grLasso), which enforces sparsity at the group level by selecting or excluding entire feature families, thereby enhancing model interpretability and stability in the presence of multicollinearity. Features with nonzero coefficients at the optimal penalty parameter were considered selected (yielding a total of five radiomic predictors in the final radiomic model), and their robustness was further characterized by their frequency of selection across cross-validation folds as well as their average estimated coefficients. Figure 2 depicts the relative magnitude and direction of the estimated mean feature coefficients, highlighting consistent differences in feature selection patterns and effect sizes. These results indicate that the choice of regularization strategy strongly influences the derived radiomic signature, with direct implications for interpretability, biological relevance, and downstream clinical inference. Table 2 illustrates the selection frequency and mean coefficients of the radiomics features, highlighting those that were consistently retained across all five cross-validation folds by the group-penalized Cox model. In addition to the radiomic model, the clinical Cox model included three predictors (overall stage, T stage, and N stage), while the demographic model comprised two predictors (age and gender). As summarized Table 3, the radiomics, clinical, and demographic models included 5, 3, and 2 predictors, respectively, whereas the fusion models were based on three modality-specific risk scores. With 351 observed events, the corresponding events-per-variable (EPV) values ranged from approximately 70 to 176, substantially exceeding recommended thresholds and supporting model stability.

### 4.2. Performance Comparison

Model performance was evaluated based on patients’ risk discrimination (C-index and mean time-dependent AUC across multiple time points) and overall prediction error (integrated Brier score, IBS). Performance was first assessed on the full dataset and then using 5-fold cross-validation to obtain a more robust estimate of out-of-sample predictive performance (Table 4 and Table 5).

As shown in Table 4, among the unimodal Cox models, the Radiomics unimodal model achieved the highest discriminative performance on the full dataset (C-index = 0.599; AUC = 0.646; IBS = 0.203), outperforming the Clinical unimodal model (C-index = 0.561; AUC = 0.589; IBS = 0.209) and the Demographics unimodal model (C-index = 0.549; AUC = 0.571; IBS = 0.213). In 5-fold cross-validation, performance decreased across all unimodal models, although the Radiomics model remained the best-performing unimodal approach (C-index = 0.5717; AUC = 0.5891; IBS = 0.209). This indicates that radiomic features provide greater prognostic separation than clinical or demographic variables alone, although the magnitude of this advantage was reduced under cross-validated evaluation.

Based on Table 5, all late fusion models demonstrated improved discrimination relative to unimodal approaches. In the full-dataset analysis, the RSF fusion model yielded the highest overall discrimination (C-index = 0.705; AUC = 0.785; IBS = 0.164), representing a substantial improvement over the Cox Fusion model (C-index = 0.614; AUC = 0.667; IBS = 0.199), Logistic Stack fusion model (C-index = 0.619; AUC = 0.676; IBS = 0.198), and Weighted Average fusion model (C-index = 0.611; AUC = 0.664; IBS = 0.200). However, in the 5-fold cross-validated analysis, performance differences were smaller, and the Cox Fusion model achieved the best overall cross-validated performance (C-index = 0.5769; AUC = 0.5994; IBS = 0.2096). Notably, under 5-fold cross-validation, the Cox Fusion model outperformed the Radiomics unimodal model, indicating that integrating multimodal information provides more robust generalizable performance than radiomic features alone.

These findings indicate that multimodal integration enhances risk ranking accuracy. However, the cross-validated analysis provides a more conservative and more reliable estimate of generalizable performance, suggesting that the improvement from multimodal fusion is more modest than indicated by the full-dataset analysis. The results presented in the remaining sections are based on 5-fold cross-validation.

### 4.3. Risk Groups

To evaluate clinical relevance, individual absolute risk estimates at the cohort’s median survival time were used to stratify patients into three prognostic groups: low, medium, and high risk. Continuous risk scores were discretized using a data-driven, outcome-guided procedure, in which candidate cutoff pairs were assessed via the log-rank statistic. In the 5-fold cross-validation framework, cutoff selection was performed exclusively within the training set of each fold. For each training fold, optimal cutoff pairs were determined using the log-rank test while enforcing minimum group sizes. The fold-specific optimal cutoffs were subsequently averaged across folds, and these mean cutoff values were applied to the test sets to assign patients to low-, medium-, and high-risk groups. This procedure ensured that risk stratification was derived independently of the test data. In addition, we have included Table 6 summarizing the number of patients, events, and event rates across risk groups for each model. This provides a clearer view of group distribution, outcome separation, and the stability of risk stratification. Although censoring is present, the consistent and monotonic separation of event rates across risk groups indicates that risk stratification remains stable and is unlikely to be driven by differential censoring patterns.

### 4.4. Risk Stratification for Baseline Models (Unimodal)

**Figure 3:**
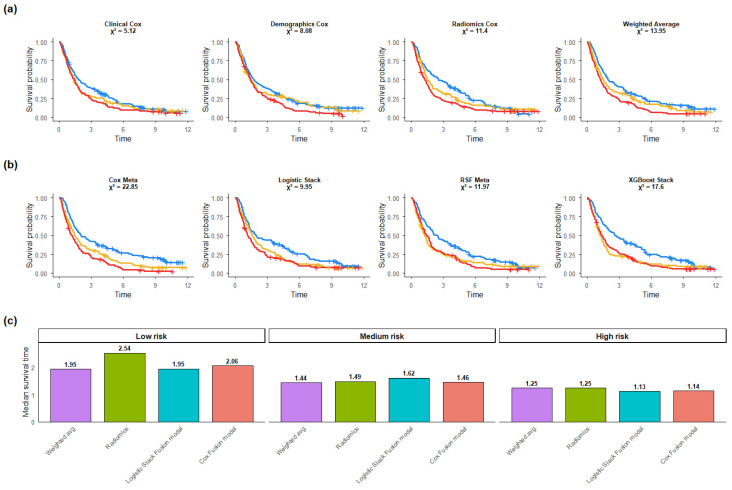
(**a**) Kaplan–Meier curves for unimodal Cox models and (**b**) late-fusion models, stratified into low-, medium-, and high-risk groups (blue = low risk group, yellow = medium risk group, red = high risk group). (**c**) Median observed survival times across risk groups.

Applying the outcome-guided stratification to unimodal models revealed monotonic separation in Kaplan–Meier survival curves across all modalities (Figure 3a). In the 5-fold cross-validation analysis, among unimodal models, the Radiomics Cox model demonstrated the strongest survival separation (χ^2^ = 11.4), followed by the Demographics Cox model (χ^2^ = 8.08) and the Clinical Cox model (χ^2^ = 5.12).

### 4.5. Risk Stratification in Late-Fusion Models

When applied to late-fusion models, cross-validated risk stratification showed improved separation for several multimodal approaches (Figure 3b). The Cox Meta model demonstrated slightly better performance among fusion models with log-rank statistic (χ^2^ = 22.85), followed by XGBoost Stack (χ^2^ = 17.6), Weighted Average (χ^2^ = 13.95), and RSF Meta (χ^2^ = 11.97). These findings suggest that multimodal integration improves prognostic stratification.

### 4.6. Median Survival-Based Comparison

Figure 3c summarizes median observed survival across risk groups for the displayed models. Across all models, median survival decreased from low- to medium- to high-risk categories, supporting the consistency of the risk-group definitions. The magnitude of separation differed across approaches.

### 4.7. Distribution of Patients Across Risk Groups

**Figure 4:**
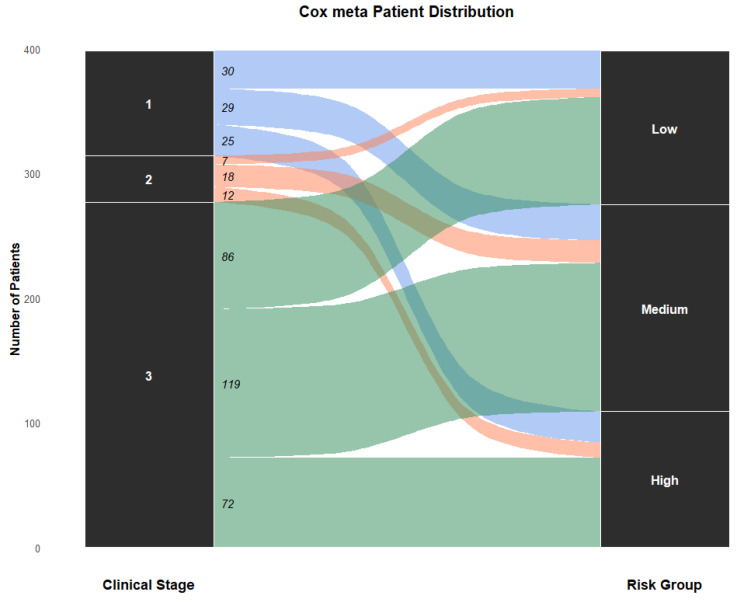
Patient distribution across clinical stages and model-derived risk groups for Cox fusion.

**Figure 5:**
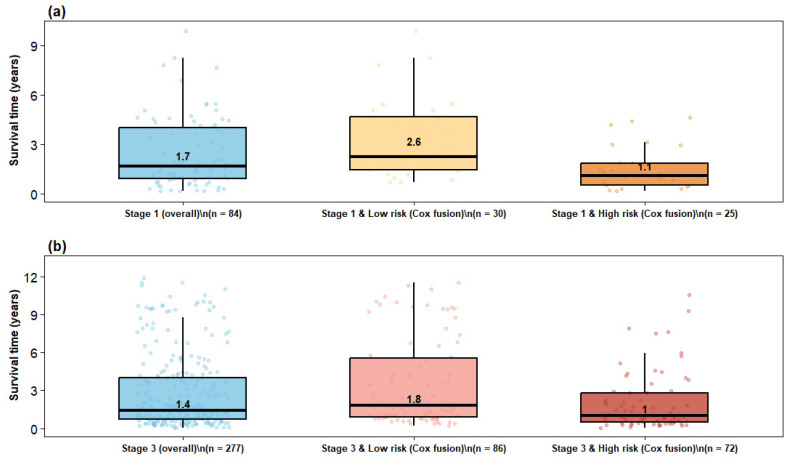
Observed survival distributions across stage and model-derived risk groups. (**a**) Stage I patients classified as high risk; (**b**) Stage III patients classified as low risk. RSF fusion shows stronger enrichment at both prognostic extremes.

To evaluate clinical interpretability, patient redistribution from conventional clinical stages to model-derived risk groups was examined using the Cox meta model (Figure 4). The Sankey diagram illustrates flows from clinical stages (I–III) to low-, medium-, and high-risk categories, showing that risk assignment was not merely reflective of stage and suggesting that multimodal fusion captures additional prognostic information beyond anatomical classification. Redistribution was especially notable within Stage I and Stage III populations, which exhibit substantial outcome heterogeneity. Post hoc survival analyses further quantified these patterns (Figure 5). Among Stage I patients, the overall cohort showed a median survival of 1.7 years, whereas the corresponding Stage III overall cohort showed a lower median survival of 1.4 years. Within the model-defined favorable groups, Stage I low-risk patients had a median survival of 2.6 years compared with 1.8 years for Stage III low-risk patients, indicating that the low-risk designation captured more prolonged survival in early-stage disease while still identifying a relatively favorable subgroup among advanced-stage patients.

A similar pattern was observed in the high-risk strata. Stage I high-risk patients had a median survival of 1.1 years, which was slightly higher than the 1.0-year median observed in Stage III high-risk patients, but both groups showed compressed survival distributions and substantially worse outcomes than their corresponding low-risk groups. This suggests that the Cox meta model enriched for adverse prognosis within both stages, while preserving the expected survival gradient between earlier and more advanced disease. More broadly, the figure shows that Stage I high-risk patients experienced survival outcomes closer to those of Stage III patients than to Stage I low-risk patients, underscoring clinically meaningful within-stage heterogeneity. Conversely, Stage III low-risk patients demonstrated an upward shift in survival relative to the overall Stage III cohort, with clearer separation from the Stage III high-risk subgroup. Together, these findings support the superior ability of the Cox meta model to stratify patients beyond conventional clinical stage and to identify both poor-prognosis patients within early-stage disease and favorable-prognosis patients within advanced-stage disease.

### 4.8. Calibration Plot Results

**Figure 6:**
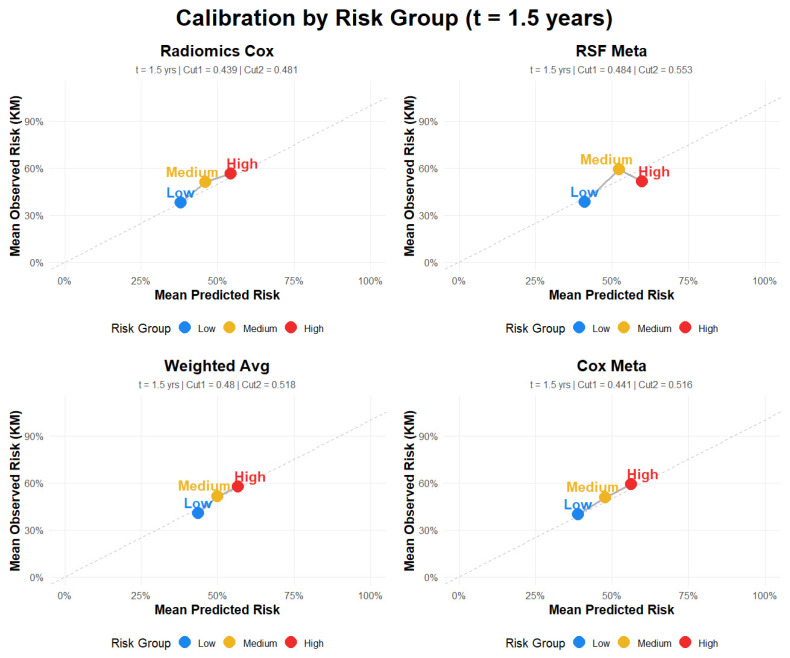
Calibration plots for fusion models.

To complement the discrimination and prediction error analyses, calibration of the predicted 1.5-year absolute risks was assessed for the risk-group–based models (Figure 6; Table 7). Calibration was evaluated using group-level calibration plots, in which mean predicted risk was compared with Kaplan–Meier–based observed risk within each prognostic group, together with calibration slope and intercept as quantitative summaries of agreement. The Cox Meta model showed the most favorable calibration profile overall, with a slope of 1.10 and an intercept of −0.027, followed closely by the Radiomics Cox model (slope = 1.12, intercept = −0.031) (Table 7). In contrast, the RSF Meta model showed evidence of weaker calibration (slope = 0.796, intercept = 0.088), and the Weighted Average model demonstrated the greatest departure from ideal calibration (slope = 1.3, intercept = −0.151). The calibration plots at 1.5 years were broadly consistent with these metrics, indicating closer agreement between predicted and observed risk for the Cox Meta and Radiomics Cox models and greater deviation for the RSF Meta and Weighted Average models. Overall, these findings indicate that the proposed risk stratification framework provides not only prognostic discrimination but also reasonably reliable absolute risk estimates for clinical interpretation at 1.5 years.

**Figure 7:**
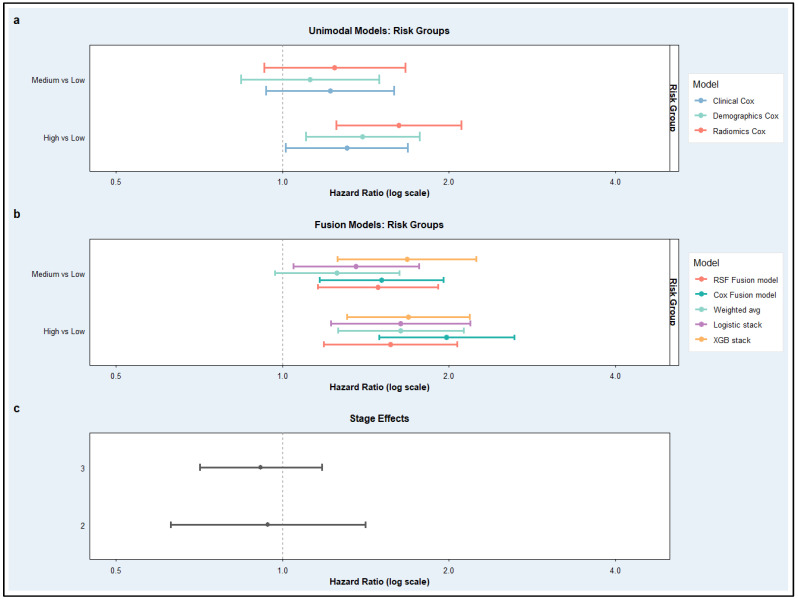
Hazard ratios associated with model-derived risk groups and clinical stage across baseline and late-fusion survival models. (**a**) Forest plot of hazard ratios (HRs) with 95% confidence intervals for baseline unimodal Cox models (Clinical, Demographics, and Radiomics). (**b**) Corresponding estimates for late-fusion models. Hazard ratios are displayed on a logarithmic scale, with the vertical dashed line indicating HR = 1 (no effect). (**c**) stage effects, showing hazard ratios for Stage II and Stage III relative to the Stage I reference group.

These improvements in predictive performance translated into clearer and more clinically meaningful risk stratification under the 5-fold cross-validation framework, as illustrated in Figure 7. Figure 7a presents hazard ratios estimated from the unimodal Cox models, showing the association of model-derived risk groups with survival outcomes. Across baseline models, medium- and high-risk groups demonstrated increased hazards relative to the low-risk reference category; however, the effect sizes were relatively modest, and confidence intervals showed greater overlap, indicating weaker separation between prognostic strata. Among the unimodal approaches, the Radiomics Cox model showed somewhat stronger differentiation between risk groups than the Clinical and Demographics Cox models. In contrast, Figure 7b displays hazard ratios derived from the late-fusion models and demonstrates substantially stronger and more coherent stratification. Compared with Figure 7a, fusion approaches produced larger hazard ratios and clearer stepwise increases from medium- to high-risk groups, although some overlap in confidence intervals remained, indicating improved separation of prognostic categories independent of clinical stage. Among the fusion strategies, the Cox Fusion model exhibited the clearest divergence between risk groups, particularly for high- versus low-risk comparisons, while the remaining fusion models also showed directionally consistent separation.

Figure 7c further contextualizes these findings by illustrating the effect of clinical stage alone, where higher tumor stages were consistently associated with elevated risk; however, the magnitude of these effects remained comparatively modest and showed overlap across stages. This highlights that, while stage retains prognostic value, it does not achieve the same level of risk discrimination as the model-derived stratifications, particularly those obtained from fusion approaches. Direct comparison of panels (a), (b), and (c) therefore indicates that multimodal late fusion not only strengthens statistical associations with survival but also yields more distinct and clinically interpretable risk separation beyond that achieved by unimodal Cox models or clinical staging alone under cross-validated evaluation, although the magnitude of improvement was more moderate than that suggested by the original full-dataset results.

## 5. Discussion

This study addressed the critical need for individualized prognostic assessment in non-small cell lung cancer (NSCLC) by developing a multimodal late-fusion framework that integrates radiomic, clinical, and demographic information. Across the analyses, multimodal integration improved predictive performance relative to unimodal approaches, supporting the complementary value of heterogeneous patient data. In contrast to our initial full-cohort analysis, however, the 5-fold cross-validation results indicated that these gains were more modest than originally suggested. Within this stricter evaluation framework, the Cox fusion model achieved the most favorable overall balance of discrimination, prediction error, and calibration, while the radiomics Cox model remained the strongest unimodal predictor. Radiomic features demonstrated independent prognostic value beyond conventional clinical and demographic variables. The radiomics-only model consistently outperformed the clinical-only and demographics-only models, supporting the premise that quantitative imaging captures tumor heterogeneity not fully reflected by standard factors alone. To address the high dimensionality and strong inter-feature correlations of radiomic data, a group Lasso–penalized Cox framework was employed, and the same five radiomic features were selected across all five folds. This stability strengthens the interpretability of the derived radiomic signature and supports its reproducible prognostic relevance.

Among the fusion models, RSF demonstrated slightly better performance using the entire dataset. However, by cross-validation assessment, Cox-based fusion approaches demonstrated more favorable and stable performance. In this setting, the unimodal radiomics Cox model showed the strongest stratification among the baseline models. However, multimodal fusion models demonstrated moderate improvement in comparison with unimodal models for patients’ risk prediction. Particularly, in addition to improved predictive performance, the fusion models also yielded more distinct risk stratification, with better separation between low-, medium-, and high-risk groups. Calibration analysis at 1.5 years provided an additional perspective, indicating that the Cox fusion model and Radiomics Cox model produced the most reliable absolute risk estimates.

These findings suggest that the proposed framework contributes not only to prognostic discrimination but also to interpretable risk estimation. Overall, the results support multimodal prognostic modeling as a promising approach in NSCLC, while underscoring the importance of rigorous validation and cautious interpretation. The integration of radiomic, clinical, and demographic information improved survival prediction and risk stratification compared with unimodal modeling; however, these gains may be modest in small to moderate-sized datasets when evaluated under strict cross-validation and should be interpreted in the context of cohort size and the internal validation design. While internal cross-validation supports the robustness of the findings, inclusion of an external validation cohort would further strengthen the generalizability of the results. Future work will be conducted to apply the proposed framework on larger cancer cohorts with potentially additional molecular or treatment-related factors.

## 6. Conclusions

This study proposed a rigorous multimodal late-fusion framework for predicting overall survival in non-small cell lung cancer (NSCLC) using imaging biomarkers, clinical, and demographic information. By integrating these complementary data sources, the proposed framework improved prognostic assessment in comparison with unimodal models, with the cross-validated analyses showing modest but consistent gains in discrimination and risk stratification. Among the evaluated models, the Cox fusion approach demonstrated the most favorable overall balance of discrimination, prediction error, and calibration, while the radiomics Cox model remained the strongest unimodal predictor. These findings support the premise that quantitative imaging captures prognostically relevant tumor heterogeneity that are not fully reflected by conventional clinical variables alone.

Beyond statistical performance, the proposed framework provides interpretable absolute risk estimates and meaningful separation of prognostic risk groups, supporting its potential relevance for individualized survival assessment in NSCLC. Overall, the findings highlight multimodal late-fusion modeling as a promising strategy for precision oncology, while emphasizing the importance of rigorous cross-validated evaluation to obtain reliable estimates of performance, calibration, and generalizability. Future work will evaluate this framework in larger external cohorts to further assess its applicability in more personalized and biologically informed oncology settings.

## Figures and Tables

**Table 1:** Baseline clinical characteristics of the study cohort.

Characteristic	Value
Number of patients (N)	398
Age, mean ± SD (years)	68.1 ± 10.1
Age, median (IQR in years)	68.7 (61.3–75.9)
Gender: Male, n (%)	273 (68.6%)
Gender: Female, n (%)	125 (31.4%)
Age group: Young (≤median), n (%)	199 (50.0%)
Age group: Old (>median), n (%)	199 (50.0%)
Stage I, n (%)	84 (21.1%)
Stage II, n (%)	37 (9.3%)
Stage III, n (%)	277 (69.6%)
Survival time, median (range in years)	1.50 (0.03–11.86)
Event status: Death, n (%)	351 (88.2%)
Event status: Censored, n (%)	47 (11.8%)

**Table 2:** Selection Frequency and Mean Coefficients of Radiomics Features.

Feature	Coefficient	Selection Frequency
**Strength**	−0.0503055	**5**
**Busyness**	0.0498012	**5**
**Complexity**	0.028127	**5**
**Contrast**	0.00419581	**5**
**Coarseness**	−0.0014766	**5**
Mesh Volume	−24.45698822	1
Voxel Volume	24.40109668	1
Surface Area	0.178487279	1
Maximum2DDiameterColumn	−0.129661808	1
Minor Axis Length	0.090214667	1
Major Axis Length	0.082821366	1
Elongation	−0.066041641	1
Least Axis Length	−0.061502877	1
Maximum 2D Diameter Slice	−0.054625188	1
Maximum 2D Diameter Row	−0.048207507	1
Surface Volume Ratio	−0.043304861	1
Sphericity	0.042185152	1
Flatness	0.029738509	1
Maximum3DDiameter	0.02578915	1

**Table 3:** Number of predictors and events used in each model, with corresponding events-per-variable (EPV) values.

Model Type	Predictors Included	Number of Predictors	Number of Events	EPV
Radiomics Cox	Selected radiomic features	5	351	70.2
Clinical Cox	Overall stage, T stage, N stage	3	351	117
Demographic Cox	Age, Gender	2	351	175.5
Fusion Models	Radiomics risk score, Clinical risk score, Demographic risk score	3	351	117

**Table 4:** Predictive performance of unimodal Cox models. Bold values indicate the best performance within each column.

Model	Whole Data Set			Cross Validated		
	C-Index	AUC	IBS	C-Index (95% CI)	AUC (95% CI)	IBS (95% CI)
Radiomics (Cox)	**0.599**	**0.646**	**0.203**	**0.5717** [0.5698, 0.5736]	**0.5891** [0.5862, 0.5920]	**0.209** [0.2087, 0.2093]
Clinical (Cox)	0.561	0.589	0.209	0.535 [0.5332, 0.5368]	0.5337 [0.5311, 0.5363]	0.2152 [0.2149, 0.2155]
Demographics (Cox)	0.549	0.571	0.213	0.5392 [0.5255, 0.5529]	0.551 [0.5469, 0.5550]	0.2903 [0.2900, 0.2906]

**Table 5:** Predictive performance of Fusion models. Bold values indicate the best performance within each column.

Model	Whole Data Set			Cross-Validated		
	C-Index	AUC	IBS	C-Index (95% CI)	AUC (95% CI)	IBS (95% CI)
RSF Fusion/Meta	**0.705**	**0.785**	**0.164**	0.5637 [0.5617, 0.5657]	0.5865 [0.5841, 0.5889]	0.2103 [0.2099, 0.2107]
Cox Fusion/Meta	0.614	0.667	0.199	**0.5769** [0.5756, 0.5782]	0.5994 [0.5972, 0.6016]	**0.2096** [0.2094, 0.2099]
Logistic Stack	0.619	0.676	0.198	0.5643 [0.5621, 0.5665]	0.597 [0.5936, 0.6004]	0.2467 [0.2464, 0.2470]
Weighted Average	0.611	0.664	0.2	0.5693 [0.5680, 0.5705]	**0.5961** [0.5945, 0.5978]	0.2109 [0.2107, 0.2111]
XGB Stack	0.691	0.776	0.209	0.5619 [0.5600, 0.5638]	0.5904 [0.5875, 0.5933]	0.2458 [0.2453, 0.2463]

**Table 6:** Distribution of patients, events, and event rates across low-, medium-, and high-risk groups for each model.

Model	Risk Group	Number of Patients	Number of Events	Event Rate
Radiomics (Cox)	Low	116	100	0.862
	Medium	97	83	0.856
	High	185	168	0.908
Clinical (Cox)	Low	170	145	0.853
	Medium	85	72	0.847
	High	143	134	0.937
Demographics (Cox)	Low	193	164	0.85
	Medium	101	90	0.891
	High	104	97	0.933
RSF Fusion/Meta	Low	139	118	0.849
	Medium	154	135	0.877
	High	105	98	0.933
Cox Fusion/Meta	Low	123	98	0.797
	Medium	166	149	0.898
	High	109	104	0.954
Weighted Average	Low	136	114	0.838
	Medium	135	117	0.867
	High	127	120	0.945
Logistic Stack	Low	109	91	0.835
	Medium	168	152	0.905
	High	121	108	0.893
XGB Stack	Low	123	101	0.821
	Medium	100	89	0.89
	High	175	161	0.92

**Table 7:** Calibration metrics.

Model	Slope	Intercept
Radiomics Cox	1.12	−0.031
RSF Fusion Model	0.796	0.088
Cox Fusion Model	1.10	−0.027
Weighted Avg	1.3	−0.151

## Data Availability

Data is publicly available and cited in the manuscript. Data Cite: NSCLC-RADIOMICS: The Cancer Imaging Archive (TCIA). Available online: https://www.cancerimagingarchive.net/collection/nsclc-radiomics/ (accessed on 23 June 2025).

## Abbreviations

NSCLC	Non-Small Cell Lung Cancer
TNM	Tumor-Node-Metastasis
ML	Machine Learning
AI	Artificial Intelligence
LASSO	Least Absolute Shrinkage and Selection Operator
CT	Computed Tomography
Cox PH	Cox Proportional Hazards
KM	Kaplan–Meier
TCIA	The Cancer Imaging Archive
